# Effects of regular consumption of a β-glucan-rich oyster mushroom powder on cholesterol metabolism in adults with moderately elevated LDL-cholesterol concentrations: a double-blind randomized controlled trial

**DOI:** 10.1186/s12986-026-01122-3

**Published:** 2026-05-03

**Authors:** Jana Johnen, Julia Waizenegger, Jörg Ellinger, Dieter Lütjohann, Kristina E. Steffens, Karl G. Wagner, Birgit Stoffel-Wagner, Ramona Dolscheid-Pommerich, Sabine Ellinger

**Affiliations:** 1 Faculty of Agricultural, Nutritional and Engineering Sciences, Institute of Nutritional and Food Science, University of Bonn, Human Nutrition, Bonn, Germany; 2 Faculty of Medicine, Department of Urology and Pediatric Urology, University of Bonn, Bonn, Germany; 3 University Hospital Bonn, Institute for Clinical Chemistry and Clinical Pharmacology, University of Bonn, Bonn, Germany; 4 Department of Pharmaceutical Technology and Biopharmacy, Pharmaceutical Institute, University of Bonn, Bonn, Germany; 5 Institute of Nutritional and Food Science, Human Nutrition, University of Bonn, Käthe-Kümmel-Str. 1, 53115 Bonn, Germany

**Keywords:** Cholesterol metabolism, Hypercholesterolemia, Noncholesterol sterols, Oyster mushrooms, β-glucans, LDL-cholesterol, Lipids, Ergosterol, Cholesterol absorption, Cholesterol synthesis

## Abstract

**Background:**

Oyster mushrooms (*Pleurotus ostreatus*, PO) are rich in β-glucans and other ingredients with cholesterol-lowering potential. While human intervention studies suggest that PO intake may reduce total cholesterol (TC), low-density lipoprotein cholesterol (LDL-C), and triglycerides, current evidence remains limited due to methodological limitations of the studies. Thus, this study investigated whether regular intake of PO powder affects LDL-C concentrations in adults with moderately elevated LDL-C (primary aim). Moreover, the study explored the effect on other lipids (TC, high-density lipoprotein cholesterol, triglycerides), on apolipoproteins A1 and B and possible underlying mechanisms of action (secondary aims).

**Methods:**

In a double-blind, randomized controlled trial, 46 adults (37 female, 9 male) with moderately elevated LDL-C (116–190 mg/dL) consumed a beverage containing 8.4 g PO powder providing 3 g of β-glucans or a beverage without PO daily over 4 weeks. Plasma concentrations of LDL-C, other lipids and apolipoproteins were measured before and after intervention. The concentrations of noncholesterol sterols in serum, normalized to cholesterol, were determined as validated surrogate markers for cholesterol absorption (sitosterol, campesterol, and 5α-cholestanol), cholesterol synthesis (lathosterol), and bile-acid synthesis (7α-hydroxycholesterol), along with ergosterol, a fungal-specific sterol. Expression of selected target genes involved in cholesterol metabolism was analyzed in blood. Statistical analysis included comparisons of the changes between the groups (treatment effect) and linear modeling.

**Results:**

PO treatment did not modulate LDL-C; no treatment effect was observed for other lipids, apolipoproteins or gene expression (*P* ≥ 0.05 for all). However, after adjustment for sex, linear model analysis showed a reduction in markers of cholesterol absorption, especially in females (*P* < 0.05 for all). No effects were observed on markers of cholesterol and bile-acid synthesis (*P* ≥ 0.05 for all). Ergosterol was detectable in all serum samples after PO intake, confirming high compliance with PO treatment.

**Conclusions:**

Daily consumption of 8.4 g of PO powder over 4 weeks has no impact on LDL-C concentrations in adults with moderately elevated LDL-C concentrations. However, post-hoc analysis indicates a sex-dependent reduction in cholesterol absorption by PO consumption, especially in females, suggesting that PO may have the potential to beneficially modulate cholesterol metabolism.

**Trial registration:**

Registration at German Clinical Trials Register; DRKS-ID: DRKS00033943; registration date: 21/03/2024. https://drks.de/search/de/trial/DRKS00033943.

**Supplementary Information:**

The online version contains supplementary material available at 10.1186/s12986-026-01122-3.

## Background

Atherosclerotic cardiovascular diseases (ASCVD) remain the leading causes of death worldwide. An improvement in the lipid profile, particularly a reduction in low-density lipoprotein cholesterol (LDL-C), is desirable owing to the causal relationship between increased LDL-C and the risk of ASCVD. Ultimately, optimizing diet and lifestyle factors forms the basis for ASCVD prevention [1].

Edible mushrooms like oyster mushrooms (*Pleurotus ostreatus*, PO) have been consumed by humans for centuries [2]. These mushrooms have a low energy density and fat content and contain high amounts of fibers, especially β-glucans [2, 3]. According to the European Food Safety Authority (EFSA), ingestion of at least 3 g/d of β-glucans from oats can reduce blood LDL-C [4]. This effect has been attributed to the increased viscosity of the chyme, formation of a gel on the intestinal mucosal surface, and binding of bile acids, which consequently lowers the re-/absorption of cholesterol and bile acids. The loss of bile acids from the enterohepatic circulation increases their *de novo* synthesis from serum cholesterol, thereby reducing serum cholesterol concentration [5]. Another mechanism proposed is the inhibition of *de novo* cholesterol synthesis by short-chain fatty acids, especially propionic acid, produced via microbial fermentation of β-glucans [6]. Even though mushroom β-glucans present a different structure [β-(1, 3)-D-glucans with short β-(1, 6)-linked side chains] compared to cereal β-glucans [linear β-(1, 3) and β-(1, 4) linked glucose residues] [7], mechanisms on cholesterol-lowering effects are thought to be similar. In vitro and animal studies have already demonstrated the beneficial effects of β-glucans from edible mushrooms on the cardiometabolic system [8].

Aside from β-glucans, other ingredients in edible mushrooms may contribute to the reduction in LDL-C. Ergosterol may lower cholesterol absorption by displacing cholesterol from the micelles [9] and cholesterol synthesis by inhibiting 24-dehydrocholesterol reductase and 7-dehydrocholesterol reductase as shown in vitro [10, 11]. Moreover, mevinolin, which can be produced from PO, acts as a competitive inhibitor of 3-hydroxy-3-methylglutaryl coenzyme A reductase, the key regulatory enzyme of the cholesterol synthesis pathway [12, 13].

A systematic review of intervention studies on the effects of PO on cardiometabolic parameters suggests that PO consumption might exert beneficial effects on lipid metabolism [14]: Various controlled intervention studies have observed a reduction in total cholesterol (TC) [15, 16], LDL-C [15], and triglycerides (TG) [15–17] in adults with type 2 diabetes [15, 16] or moderate untreated hyperlipidemia [17] after regular PO intake at doses of 30 g/d [17], 150 g/d [16], 200 g/d [15] for 1 week [16], 3 weeks [17] and 1 year [15]. However, owing to methodological limitations (e.g., the lack of randomization and blinding) and high risk of bias, evidence supporting the beneficial effects of PO intake on the lipid profile remains weak [14]. Systematic reviews considering intervention studies on cardiometabolic benefits of edible mushroom consumption in general also conclude that potential effects are inconsistent but weak evidence suggests lipid lowering potential [18–20].

Therefore, this double-blind randomized controlled trial (RCT) aimed to investigate whether the regular consumption of a β-glucan-rich PO powder could affect the concentrations of LDL-C in adults with moderately elevated LDL-C concentrations as primary prevention for subjects with low cardiovascular risk (primary aim). Moreover, the study aimed to explore the effect on other lipids (TC, high-density lipoprotein cholesterol [HDL-C], and TG), apolipoprotein A1 (ApoA1), and apolipoprotein B (ApoB) and underlying possible mechanisms (secondary aims).

## Methods

This trial was conducted between 07/2024 and 11/2024 at the Institute of Nutritional and Food Science, Human Nutrition, University of Bonn, Germany after approval by the Ethics Committee of the University of Bonn (project ID: 443/23-EP; date of approval: 07/03/2024) and registration at the German Clinical Trials Register (DRKS-ID: DRKS00033943; date of registration: 21/03/2024). Written informed consent was obtained from all participants before inclusion. This study was performed in accordance with the principles stated in the Declaration of Helsinki. This manuscript was drafted following the Consolidated Standards of Reporting Trials (CONSORT) statement [21].

### Study population

Participants were recruited between 04/2024 and 09/2024 through in-house postings at the University of Bonn and through public announcements in the Bonn area (newsletters, newspapers, flyers, and social media posts). The inclusion criteria were as follows: age ≥ 18 years, nonsmoking and moderately elevated LDL-C concentrations [116–190 mg/dL according to the ESC/EAS guidelines for the management of dyslipoproteinemia [1]]. The exclusion criteria were as follows: known gastrointestinal diseases, any disease or medication that might affect the endpoints including lipid-lowering agents, allergies/intolerances to the granule ingredients, intake of phytosterol-enriched foods and supplements providing β-glucans or other soluble fibers, participation in another clinical trial (currently or within the past month), planned changes in lifestyle as well as pregnancy and lactation. The eligibility criteria were checked using a questionnaire except in two circumstances: [1] subjects with interest to participate in the present study were screened for moderately elevated fasting LDL-C concentrations if no premedical diagnosis on moderately elevated LDL-C concentrations was available from the past 3 months and [2] a urine-based pregnancy test was additionally performed in premenopausal women to exclude pregnancy (ACCU-TELL HCG rapid pregnancy test, AccuBiotech Co., Beijing, China).

### Study design and intervention

This double-blind, parallel group RCT included participants who consumed a beverage based on a granule providing either 8.4 g powdered PO or without PO (placebo) daily for 4 weeks. After enrollment by the medical advisor, participants were allocated to either group A or group B depending on the kind of treatment received (PO vs. placebo) using permuted block randomization (ratio 1:1, block size of 4; Sealed Envelope, Sealed Envelope Ltd., London, United Kingdom) by another person outside the research staff. The allocation sequence was generated by a technician not involved in data analysis. Participants were assigned to the groups in the order of recruitment using an Excel spread sheet to ensure allocation concealment. The research staff remained blinded until statistical analysis had been finished.

Investigations were performed in our study center before and after the 4-week intervention after at least a 12-h overnight fast. Anthropometric measurements were obtained, and venous blood samples were collected. Compliance with lifestyle instructions (see below) was determined based on guided interviews and standardized food records conducted for 3 days before both visits.

To ensure comparable conditions on both visits, participants were instructed to consume a similar dinner until 7:00 pm on both previous days and to refrain from alcohol consumption and any extraordinary physical activities. Premenopausal women were investigated at the same menstrual cycle phase to exclude potential effects on outcome parameters [22, 23].

### Composition and preparation of the test beverages

The test beverages were prepared using granules containing either PO or no PO (placebo). A daily portion of the granule with 8.4 g PO powder provided 3.0 g of β-glucans (determined photometrically using β-glucan Yeast & Mushroom Assay Kit K-YBGL [Megazyme Int., Bray, Ireland]) and 5.34 mg total ergosterol (determined by gas chromatography-coupled mass spectrometry [GC-MS] with 23.8% free and 76.2% esterified ergosterol).

Both granules were produced by the Pharmaceutical Institute, Department of Pharmaceutical Technology and Biopharmacy, University of Bonn using dry granulation with a roller compactor (MINI POLYGRAN^®^ 150/30/3, Gerteis Maschinen + Processengineering, Jona, Switzerland). Table 1 details the composition of a daily portion of each granule. To ensure blinding, iced tea peach flavor was added to standardize the taste and smell of the beverage. Both granules were packaged into plain sachets using a contract filler (German’s Best Packaging Service, Denzlingen, Germany) who encoded the treatments and labeled the sachets with “A” and “B.” The document showing the allocation to each treatment was kept in an opaque, sealed envelope until completion of statistical analysis.

The daily portion was provided in two sachets. The participants were instructed to dissolve the content of each sachet in at least 250 mL water immediately before consumption and to consume each beverage with a meal. According to the manufacturer of the PO powder, 8.4 g provided 2.2 g protein, 0.1 g fat, and 1.7 g carbohydrates. A daily portion of the beverage prepared from granule containing PO provided 25 kcal, whereas the placebo beverage provided no energy.

**Table 1:** Composition of the granules per daily portion

	Granule with PO	Granule without PO (Placebo)
Pleurotus ostreatus powder^a^, g	8.40	-
Microcrystalline cellulose (Vivapur 101)^b^, g	2.31	10.80
Iced peach flavor^c^, g	0.51	0.51
Citric acid^d^, g	0.31	0.31
Magnesium stearate^e^, g	0.06	0.06
Stevia^f^, g	0.02	0.02
Total weight, g	11.61	11.70

^a^ Wohlrab Pleurotus organic powder (BioFungi, Langweid am Lech, Germany) derived from oven-dried fruiting bodies providing 3 g of β-glucans determined photometrically using β-glucan (Yeast & Mushroom) Assay Kit K-YBGL (Megazyme Int., Bray, Ireland). ^b^ J. Rettenmaier & Söhne, Rosenberg, Germany. ^c^ Stockmeier Food, Stockmeier Holding, Herford, Germany. ^d^ Jungbunzlauer Ladenburg, Ladenburg, Germany. ^e^ Peter Greven, Bad Münstereifel, Germany. ^f^ Truvia Stevia RA95, Cargill Inc., Düsseldorf, Germany. PO, *Pleurotus ostreatus*

### Blood sampling and preparation

Blood samples were collected into tubes (Monovettes, Sarstedt, Nümbrecht, Germany) coated with heparin (lipids, ApoA1, and ApoB in the plasma), ethylenediaminetetraacetic acid (EDTA) (gene expression) or in tubes without anticoagulants containing a clot activator (cholesterol, noncholesterol sterols [NCS] and 7α-hydroxycholesterol in the serum). All blood tubes containing EDTA were placed on ice immediately for transport to the laboratory. Blood samples in tubes with a clot activator remained at room temperature. After at least 20 min of clotting, the samples were centrifuged (2500 ⋅ *g*, 10 min) to obtain serum. EDTA whole blood and serum samples were frozen at − 80 °C until study completion and subsequently analyzed. Fresh heparinized blood samples were transported to the Central Laboratory at the Department of Clinical Chemistry and Clinical Pharmacology, University Hospital Bonn, Germany, for analysis of lipids, ApoA1, and ApoB concentrations on the respective study days.

### Lipid status and apolipoproteins A1 and B

LDL-C, TC, HDL-C, TG, ApoA1, and ApoB concentrations were determined by the Central Laboratory at the Department of Clinical Chemistry and Clinical Pharmacology, University Hospital Bonn, Germany, accredited by the German Accreditation Body according to DIN EN ISO 15,189, in heparinized fresh plasma using enzymatic colorimetric test kits (LDLC3, Chol2, HDLC4, and TRIGL; Roche/Hitachi, Mannheim, Germany) with fully automated standardized analysis using Cobas 8000 module c702 (Roche/Hitachi). Inter-assay coefficients of variation (CV) were as follows: LDL-C, 1.53%; TC, 1.46%; HDL-C, 1.31%; and TG, 1.11%. Non-HDL-C concentrations were calculated by subtracting HDL-C from TC, after which the HDL-C/non-HDL-C ratio was calculated. ApoA1 and ApoB concentrations were determined in plasma through immunoturbidimetry using the test kits APOAT and APOBT (Roche/Hitachi), respectively, on a Cobas 8000 module c502 (Roche/Hitachi) (CV: ApoA1, 2.75%; ApoB, 2.33%). The ratio ApoB/ApoA1 was calculated.

### Noncholesterol sterols

Serum concentrations of the NCS sitosterol and campesterol (plant sterols), 5α-cholestanol (5α-saturated cholesterol metabolite), lathosterol (cholesterol precursor), and oxysterol 7α-hydroxycholesterol (cholesterol oxidation metabolite) were determined by the Laboratory for Special Lipid Analysis at the Institute for Clinical Chemistry and Clinical Pharmacology, University Hospital Bonn, as described in detail by Šošić-Jurjević et al. [24], using gas chromatography-coupled mass spectrometry in the selected-ion-monitoring mode (GC-MS-SIM). The concentrations of sitosterol, campesterol, 5α-cholestanol, and lathosterol were adjusted to serum cholesterol concentrations measured using gas chromatography coupled with a flame ionization detector (GC-FID). All measurements were expressed as a ratio to cholesterol (µg/mg cholesterol), which we herein refer to as the sitosterol, campesterol, and 5α-cholestanol ratios. These ratios have been validated as serum biomarkers of cholesterol absorption, with the lathosterol ratio having been validated as a biomarker of cholesterol synthesis [25–30]. Measurement of 7α-hydroxycholesterol, a biomarker for bile-acid synthesis, is also presented as a ratio to cholesterol (ng/mg cholesterol). The concentration of ergosterol was also quantified by the same laboratory using GC-MS (limit of detection: 0.2 µg/mL, limit of quantification: 0.5 µg/mL).

### Expression of target genes in cholesterol metabolism

The gene expression (determined as mRNA expression) of low-density lipoprotein receptor (*LDLR*), sterol regulatory element binding transcription factor 2 (*SREBF2*), 3-hydroxy-3-methylglutaryl-CoA reductase (*HMGCR*), and cytochrome P450, family 7, subfamily A, polypeptide 1 (*CYP7A1*) was determined via real-time quantitative polymerase chain reaction (RT-qPCR) at the Institute of Nutritional and Food Science, Human Nutrition, University of Bonn. The expression of glyceraldehyde-3-phosphate dehydrogenase (*GAPDH*) and β-actin (*ACTB*) as endogenous reference genes was measured.

RNA was isolated from EDTA blood using the NucleoSpin RNA Blood Kit (Macherey-Nagel, Düren, Germany). RNA quality and quantity were determined using a NanoDrop 8000 (Thermo Fisher Scientific, Waltham, MA, United States). Extracted RNA was transcribed into cDNA using the High-Capacity cDNA Reverse Transcription Kit (Thermo Fisher Scientific), after which cDNA was stored at − 80 °C until RT-qPCR analysis. Duplex RT-qPCR was performed on a Q-qPCR instrument (Quantabio, Beverly, MA, United States) using the TaqMan Multiplex Master Mix and predesigned TaqMan Gene Expression Assays (Thermo Fisher Scientific). Reactions were run in triplicate using FAM-labeled assays for *CYP7A1* (Hs00167982_m1), *SREBF2* (Hs01081784_m1), *ACTB* (Hs99999903_m1), and VIC-labeled assays for *HMGCR* (Hs00168352_m1), *LDLR* (Hs01092524_m1), and *GAPDH* (Hs99999905_m1). For the relative quantification of mRNA expression using the comparative 2^−∆∆C^_T_ method [according to Livak et al. [31]], quantification cycle (Cq) values, which were automatically determined using the integrated dynamic method of the Q-qPCR Instrument software (version 1.0.4; fluorescence cutoff at 5%), were normalized to the reference genes *GAPDH* and *ACTB* and related to the baseline values for fold-change calculation.

### Anthropometric parameters, dietary intake, physical activity, and stool type

Body weight and height, as well as waist and hip circumference, were measured under standardized conditions to calculate the body mass index (BMI) and waist-to-hip ratio, respectively [32, 33]. Body fat mass was determined through bioelectric impedance analysis at 50 kHz and 800 µA according to the ESPEN guidelines [34] using the equation of Kyle et al. [35].The participants were asked to document their food intake using standardized 3-day food records before each study day. The mean daily intake of energy and selected nutrients was calculated using DGExpert 2.0.36.1 (German Nutrition Society, Bonn, Germany) based on the official German nutrition database “Bundeslebensmittelschlüssel” (version 3.02). Physical activity was assessed using a validated International Physical Activity Questionnaire [IPAQ short form [36]] at each visit. Furthermore, participants recorded the number, amount, and form/consistency of their stools over 3 days before each visit to assess stool type using the Bristol stool chart [37].

### Compliance

The participants prospectively recorded the number of ingested serving sizes of the granule in a diary throughout the 4-week intervention. Moreover, the remaining sachets had to be returned at the second visit. Compliance was determined based on the number of serving sizes ingested in relation to the number of serving sizes that should have been consumed. The participants evaluated the taste of the test beverages at the second visit using a 5-point Likert scale, with 1 indicating “I did not like the taste at all”, 2 indicating “I did not like the taste,” 3 indicating “the taste was moderate,” 4 indicating “the taste was good” and 5 indicating “I liked the taste very much.” Compliance to lifestyle instructions was assessed based on food records and IPAQ (physical activity).

### Adverse effects

At both visits, the participants were asked about potential gastrointestinal complaints (bloating, flatulence, abdominal pain, constipation, diarrhea, liquid stools, hard stools, feeling of fullness, noticeable stomach or bowel noises, and nausea) during the past 4 weeks while considering the intensity using a 5-point Likert scale, with 1 indicating “no complaints,” 2 indicating “mild complaints,” 3 indicating “moderate complaints,” 4 indicating “severe complaints,” and 5 indicating “very severe complaints”.

### Sample size estimation

Suitable data on the effects of edible mushrooms with known β-glucan content on LDL-C concentrations have been lacking. Given our expectation of effects similar to those observed for oat β-glucans, our sample size was estimated based on the results of an RCT by Liatis et al. that utilized a double-blind parallel group design in adults (23 males, 18 females) with a LDL-C concentration ≥ 130 mg/dL who consumed either white bread enriched with oat-β-glucans (3 g) or white bread without enrichment (control) daily over 3 weeks. Fortifying the bread with oat β-glucans lowered LDL-C concentrations by − 0.66 ± 0.80 mmol/L (*P* = 0.001; mean ± SD), whereas no changes were observed in the control group (− 0.11 ± 0.45 mmol/L; mean ± SD; *P* = 0.32) [38]. Assuming a comparable effect size (d = 0.8474; according to Cohen’s dz) for oat and mushroom β-glucans on LDL-C concentrations, 23 participants per group were required to detect a statistically significant difference in the changes between both groups [calculated using G*Power 3.1.9.6, Düsseldorf, Germany [39]] using a two-sided *t*-test with an alpha error of 0.05 and a power of 80%. Assuming a dropout rate of 10%, 25 individuals per group needed to be included in the study.

### Statistical analysis

Treatment effects were determined by comparing differences (values before and after intervention) between the two groups using Student’s *t*-test for unpaired samples. If the differences were not normally distributed according to the Shapiro–Wilk test, the corresponding 95% confidence intervals (CIs) were determined by bootstrapping. Data for NCS to cholesterol ratios were first logarithmized. Nominal data were compared using the χ^2^ test. Should the later not be applicable, Fisher’s exact test was used.

Linear models (LM) were applied to examine the effects of the treatment on lipids, ApoA1, ApoB, and NCS, with treatment as a fixed effect. Furthermore, the impact of baseline values, age, sex, and BMI on LDL-C, other lipids, ApoA1, ApoB, ApoB/ApoA1, and NCS, fiber intake at baseline, as well as the interactions of treatment ⋅ sex, treatment ⋅ BMI and treatment ⋅ age, were analyzed using LM.

Metric data were presented as means ± SEM unless stated otherwise. Nominal data were provided as absolute or relative frequencies. Statistical analysis was performed using IBM SPSS Statistics version 29 (IBM Deutschland, Ehningen, Germany), with *P* values < 0.05 indicating statistical significance.

## Results

**Fig. 1:**
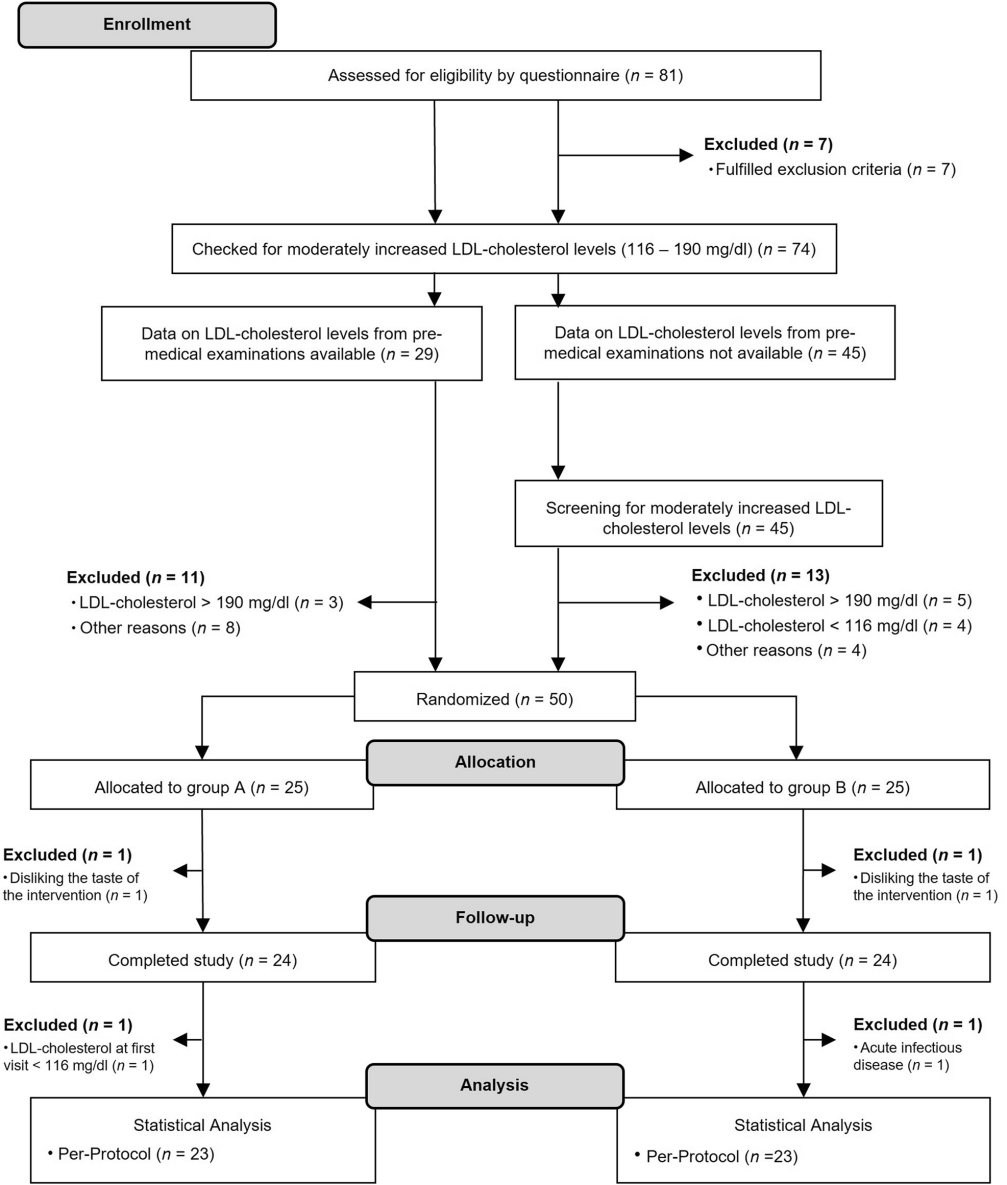
Flow diagram of participant selection

After assessing a total of 81 individuals for eligibility, 7 were excluded for meeting the exclusion criteria checked using a questionnaire. Among the 74 remaining individuals, 29 had data on LDL-C concentrations from premedical examinations (18 had values in the target range, 3 were excluded for having values above 190 mg/dL, whereas 8 with suitable values were excluded for other reasons [e.g., scheduling problems]). LDL-C concentrations were determined in 45 individuals as part of the screening procedure (32 had values in the target range, 9 were excluded for having LDL-C concentrations outside the target range, and 4 with suitable values were excluded for other reasons [e.g., scheduling problems]). Ultimately, 50 participants were included in the study and randomly allocated to either group A or B. One participant from each group dropped out for having aversions to the beverages. One participant from each group was excluded from analysis (one had LDL-C concentrations below 116 mg/dL at first visit, whereas the other had acute infectious disease at the second visit). Hence, 46 participants (9 men and 37 women [34 post- and 3 premenopausal]; age 61 ± 10 years; BMI 24.6 ± 3.6 kg/m^2^, and LDL-C concentrations 153.1 ± 23.8 mg/dL; data: means ± SD) completed the study according to the protocol and were included for statistical analyses (Fig. 1). The included participants rated the taste of both beverages as moderate (PO group, 2.8 ± 0.2; placebo group, 3.1 ± 0.2). Details regarding the baseline characteristics of the participants are presented in Table 2.

**Table 2:** Baseline characteristics of the study participants

	All (*n* = 46)	PO group (*n* = 23)	Placebo group (*n* = 23)
Sex (males/females), *n*	9/37	5/18	4/19
Age, years	61 ± 10	62 ± 5	60 ± 14
Body height, m	1.69 ± 0.08	1.70 ± 0.10	1.68 ± 0.06
Body weight, kg	70.8 ± 13.8	73.3 ± 14.2	68.2 ± 13.1
Body mass index, kg/m^2^ Body mass index classification^a^ Normal weight, *n* Overweight, *n* Obesity, class I, *n*	24.6 ± 3.6 19 11 6	25.1 ± 3.7 13 5 5	24.1 ± 3.4 16 6 1
LDL-C, mg/dL	153.1 ± 23.8	150.2 ± 25.0	156.0 ± 22.6

Data are presented as arithmetic mean ± SD unless otherwise indicated. Data refer to the first study visit. ^a^ According to WHO. LDL-C, low-density lipoprotein cholesterol; PO, *Pleurotus ostreatus*

### LDL cholesterol

No significant difference in the change in LDL-C concentrations was observed between the PO and placebo groups (Δ 4.5 mg/dL, 95% CI: −5.5, 13.1, *P* = 0.375; Table 3). LDL-C (Δ − 0.4 mg/dL, *P* = 0.029) and BMI (Δ 2.1 kg/m^2^, *P* = 0.028) at baseline were significant predictors for changes in LDL-C independent of the treatment received. LM analyses showed that sex, age, and fiber intake at baseline did not influence the effects of treatment on LDL-C.

**Table 3:** Lipid status before and after treatment with PO or placebo

	PO group (*n* = 23)			Placebo group (*n* = 23)			*P* value^a^	Treatment effect
	Week 0	Week 4	∆	Week 0	Week 4	∆		
LDL-C, mg/dL	150.2 ± 5.2	151.1 ± 5.0	1.0 ± 3.4	156.0 ± 4.7	152.5 ± 4.2	−3.6 ± 3.5	0.375	4.5 (− 5.5; 13.1)^b^
TC, mg/dL	233.2 ± 7.3	233.5 ± 6.1	0.3 ± 3.7	236.7 ± 5.1	230.0 ± 5.4	−6.8 ± 3.9	0.193	7.1 (− 3.7; 17.9)
HDL-C, mg/dL	71.4 ± 3.8	70.6 ± 3.8	−0.7 ± 1.3	68.5 ± 3.0	66.4 ± 2.8	−2.1 ± 1.2	0.442	1.4 (− 2.4; 5.0)
Triglycerides, mg/dL	118.3 ± 20.3	108.1 ± 12.8	−10.2 ± 12.0	98.6 ± 10.3	97.9 ± 10.3	−0.7 ± 6.4	0.501	−9.5 (− 36.6; 15.2)^b^
ApoA1, g/L	1.73 ± 0.06	1.72 ± 0.05	−0.01 ± 0.03	1.69 ± 0.05	1.65 ± 0.05	−0.03 ± 0.03	0.688	0.02 (− 0.07; 0.11)
ApoB, g/L	1.06 ± 0.04	1.09 ± 0.04	0.03 ± 0.02	1.11 ± 0.03	1.10 ± 0.03	−0.01 ± 0.02	0.137	0.04 (− 0.01; 0.10)
ApoA1/ApoB	0.63 ± 0.03	0.65 ± 0.03	0.02 ± 0.02	0.68 ± 0.03	0.68 ± 0.02	−0.001 ± 0.02	0.482	0.02 (− 0.03; 0.08)^b^
Non-HDL-C, mg/dL	161.8 ± 6.5	162.9 ± 5.3	1.0 ± 3.6	168.3 ± 5.3	163.6 ± 5.2	−4.7 ± 3.9	0.603	−0.01 (− 0.06; 0.03)^b^
HDL-C/non-HDL-C	0.46 ± 0.04	0.45 ± 0.03	−0.02 ± 0.02	0.42 ± 0.03	0.42 ± 0.03	−0.004 ± 0.01	0.614	−0.01 (− 0.02; 10.01)^b^

Data are presented as arithmetic mean ± SEM or mean difference (95% CI). ^a^ Comparison of the changes between the groups using unpaired Student’s *t*-test; ^b^ 95% CIs were estimated via bootstrapping. ApoA1, apolipoprotein A1; ApoB, apolipoprotein B; C, cholesterol; HDL-C, HDL cholesterol; LDL-C, LDL cholesterol; PO, *Pleurotus ostreatus*; TC, total cholesterol

### Other lipids and apolipoproteins

No significant differences in the changes in other lipids (i.e., TC, HDL-C, TG, non-HDL-C and HDL-C/non-HDL-C ratio) were observed between the PO and placebo groups (*P* ≥ 0.05 for all; Table 3). No treatment effects could be observed for ApoA1, ApoB, and ApoB/ApoA1 (*P* ≥ 0.05 for all; Table 3).

LM analysis showed that the change in ApoB/ApoA1 was influenced by the ratio of ApoB/ApoA1 at baseline (∆ −0.44, 95% CI: −0.61, − 0.11, *P* = 0.002). Changes in all lipids and apolipoproteins were not influenced by sex, age, and BMI regardless of the treatment received (*P* ≥ 0.05 for all).

### Noncholesterol sterols

No significant differences in the changes in the NCS ratios as surrogate markers for cholesterol absorption (campesterol ratio, sitosterol ratio, and 5α-cholestanol ratio), cholesterol synthesis (lathosterol ratio), and bile-acid synthesis (7α-hydroxycholesterol ratio) were observed between the PO and placebo groups (*P* ≥ 0.05 for all; Table 4).

**Table 4:** Noncholesterol sterol ratios before and after treatment with PO or placebo

	PO group (*n* = 23)			Placebo group (*n* = 23)			*P* value^a^	Treatment effect
	Week 0	Week 4	∆^b^	Week 0	Week 4	∆^b^		
Cholesterol, mg/dL^b, c^	225.4 ± 6.3	221.6 ± 5.8	−3.7 ± 4.1	244.5 ± 5.3	237.8 ± 5.2	−6.7 ± 3.8	0.588	3.0 (− 8.1; 13.3)^d^
Campesterol	1.94 (1.63; 2.32)	1.84 (1.55; 2.20)	−0.05 ± 0.03	1.63 (1.31; 2.03)	1.66 (1.35; 2.04)	0.02 ± 0.04	0.185	0.93 (0.84; 1.04)
Sitosterol	1.31 (1.09; 1.58)	1.20 (0.99; 1.45)	−0.09 ± 0.03	1.01 (0.84; 1.19)	0.97 (0.81; 1.16)	−0.04 ± 0.04	0.301	0.95 (0.86; 1.05)
5α-Cholestanol	2.06 (1.88; 2.26)	2.03 (1.83; 2.24)	−0.02 ± 0.01	2.23 (2.06; 2.42)	2.32 (2.12; 2.52)	0.04 ± 0.02	0.063	0.95 (0.90; 1.00)
Lathosterol	0.94 (0.80; 1.09)	0.92 (0.78; 1.01)	−0.02 ± 0.05	0.96 (0.83; 1.10)	0.93 (0.80; 1.10)	−0.03 ± 0.05	0.849	1.01 (0.86; 1.19)^e^
7α-Hydroxy-cholesterol^f^	15.38 (12.98; 18.22)	13.04 (10.70; 15.90)	−0.16 ± 0.07	20.22 (16.96; 24.11)	20.54 (16.94; 24.91)	0.02 ± 0.06	0.057	0.83 (0.69; 1.01)

Data are presented as geometric means (95% CI) unless otherwise indicated. All values are presented as a ratio to cholesterol (μg/mg) unless otherwise indicated. ^a^ Comparison of the changes between the groups using unpaired Student’s *t*-test; ^b^ Arithmetic means ± SEM; ^c^ cholesterolconcentrations measured via GC-FID, used to calculate the ratios; ^d^ arithmetic mean (95% CI); ^e^ 95% CI was estimated via bootstrapping; ^f^ given as a ratio to cholesterol (ng/mg). PO, *Pleurotus ostreatus*

LM analysis showed that changes in all NCS were not influenced by the corresponding baseline values. However, changes in cholesterol absorption markers campesterol ratio and sitosterol ratio were influenced by sex (campesterol ratio: Δ 0.32 µg/mg, *P* = 0.007 female vs. male; sitosterol ratio: Δ 0.20 µg/mg, *P* = 0.012 female vs. male). After adjusting for sex, a treatment effect was detected for the campesterol ratio (Δ 0.29 µg/mg, *P* = 0.043, PO vs. placebo group) and 5α-cholestanol ratio (Δ − 0.17 µg/mg, *P* = 0.002 PO vs. placebo group). Moreover, a significant treatment-by-sex interaction was found for the changes in the campesterol ratio (interaction: Δ − 0.45 µg/mg, *P* = 0.002), sitosterol ratio (interaction: Δ − 0.27 µg/mg, *P* = 0.008), and 5α-cholestanol ratio (interaction: Δ 0.14 µg/mg, *P* = 0.023). In particular, the mean campesterol ratio increased in men but decreased in women receiving PO treatment. Moreover, men and women receiving PO treatment showed a decrease in the mean 5α-cholestanol ratio, with the decrease being greater among men. Treatment with PO significantly reduced the change in the sitosterol ratio among women but not among men.

BMI at baseline was a negative predictor for changes in the sitosterol ratio (Δ − 0.04 µg/mg, *P* < 0.001). Adjusting for BMI at baseline revealed a significant treatment effect on the changes in the sitosterol ratio (Δ − 0.86 µg/mg, *P* < 0.001, PO vs. placebo group) and 5α-cholestanol ratio (Δ 0.43 µg/mg, *P* = 0.037, PO vs. placebo group). The effect on the sitosterol ratio was stronger among participants with a lower BMI than in those with a higher BMI at baseline (interaction: 0.03 µg/mg, *P* = 0.003). The difference in the 5α-cholestanol ratio between the groups decreased as BMI at baseline increased (interaction: −0.02 µg/mg, *P* = 0.015).

Changes in the 7α-hydroxycholesterol ratio or lathosterol ratio were not affected by treatment and were not influenced of sex, age, or BMI (*P* ≥ 0.05 for all). Only a significant treatment-by-age interaction was noted for the change in the 7α-hydroxycholesterol ratio (interaction: −0.03 ng/mg, *P* = 0.039). In other words, the effectiveness of PO in increasing the 7α-hydroxycholesterol ratio was reduced with age.

A reduction in the campesterol ratio was more frequently observed in the PO group than in the placebo group (*n* = 16 vs. *n* = 8, *P* = 0.019), especially in women (*n* = 14 vs. *n* = 4, *P* < 0.001) who more often experienced a reduction in the sitosterol ratio (*n* = 15 vs. *n* = 9, *P* = 0.038).

After the intervention, all participants in the PO group showed serum ergosterol concentrations above the limit of quantification (21 ± 1 µg/mL, mean ± SD), whereas serum samples from the placebo group showed no detectable ergosterol.

### Gene expression analysis

No treatment effect was observed for the mRNA expression of *LDLR*,* SREBF2*, and *HMGCR* (*P* ≥ 0.05 for all; Table 5). Age and sex did not influence the effects of treatment on the expression of these genes (*P* ≥ 0.05 for all).

**Table 5:** Changes in mRNA expression after the 4-week intervention in relation to baseline

Gene name	Gene symbol	Mean fold change		*P* value^a^	Treatment effect^b^
		PO group	Placebo group		
Low-density lipoprotein receptor	*LDLR*	1.68 (1.16; 2.44) *n* = 22	0.99 (0.59; 1.67) *n* = 21	0.106	0.76 (− 0.20; 1.67)
Sterol regulatory element binding transcription factor 2	*SREBF2*	1.49 (1.02; 1.53) *n* = 20	0.96 (0.37; 2.47) *n* = 18	0.448	0.63 (− 0.96; 2.22)
3-Hydroxy-3-methylglutaryl-CoA reductase	*HMGCR*	1.26 (0.78; 2.04) *n* = 21	0.93 (0.54; 1.62) *n* = 19	0.472	0.42 (− 0.67; 1.48)

Data are presented as geometric means (95% CI). ^a^ *P* values refer to unpaired Student’s *t*-test performed on logarithmized data; ^b^ 95% CI was estimated via bootstrapping based on logarithmized data. PO, *Pleurotus ostreatus*

Cq values for *CYP7A1* expression were not available for most samples due to extremely low expression (Cq > 40, *n* = 22; Cq 35–40, *n* = 19). Consequently, *CYP7A1* was excluded from downstream quantitative analysis.

###  Anthropometric parameters, dietary intake, physical activity, and stool type

Anthropometric parameters at both visits, dietary intake, physical activity, and stool type prior to each study day, as well as their respective changes were comparable between the groups (Table 6).

**Table 6:** Anthropometric parameters, dietary intake, physical activity, and stool characteristics before and after treatment

	PO group (*n* = 23)			Placebo group (*n* = 23)		
	Week 0	Week 4	∆	Week 0	Week 4	∆
Anthropometric parameters						
Body weight, kg	73.3 ± 14.2	72.1 ± 14.1	−1.2 ± 3.4	68.2 ± 13.1	68.6 ± 13.0	0.5 ± 3.9
Fat mass, % body weight	33.5 ± 5.8	33.3 ± 5.7	−0.2 ± 0.4	32.9 ± 6.7	33.7 ± 6.0	0.8 ± 2.4
Waist circumference, cm	88.1 ± 11.1	87.4 ± 10.3	−0.7 ± 3.4	84.5 ± 10.0	84.8 ± 10.1	0.4 ± 4.7
Waist-to-hip ratio	0.89 ± 0.05	0.88 ± 0.05	−0.003 ± 0.04	0.87 ± 0.07	0.87 ± 0.07	−0.004 ± 0.04
Dietary intake						
Energy, kcal/day	2284 ± 1150	2346 ± 883	62 ± 978	2322 ± 957	2116 ± 741	−206 ± 930
Protein, EN%/day	16 ± 3	15 ± 3	−1 ± 4	14 ± 3	14 ± 3	0.2 ± 4
Carbohydrates, EN%/day	46 ± 7	46 ± 7	0.1 ± 10	49 ± 9	50 ± 9	1 ± 8
Fat, EN%/day	40 ± 1	40 ± 8	−0.1 ± 10	38 ± 8	37 ± 9	−1 ± 8
Fiber, g/day	30 ± 16	30 ± 13	1 ± 15	28 ± 11	26 ± 12	−3 ± 10
Cholesterol, mg/day	282 ± 140	277 ± 181	−5 ± 181	369 ± 185	333 ± 199	−36 ± 215
SFA, EN%/day	15 ± 4	14 ± 2	−0.8 ± 4	15 ± 4	15 ± 5	−0.1 ± 4
MUFA, EN%/day	14 ± 4	15 ± 7	2 ± 7	13 ± 4	13 ± 4	0.1 ± 4
PUFA, EN%/day	8 ± 5	8 ± 5	0.4 ± 4	7 ± 3	7 ± 3	−0.4 ± 3
Physical activity						
MET-min/week	5439 (3047; 7212)^a^	4773 (1968; 7359)^a^	0 (− 2563; 1766)^a^	5337 (2552; 11010)^a^	7623 (3250; 10725)^a^	105 (− 3096; 4331)^a^
IPAQ-sitting, min/week	370 ± 136^a^	330 ± 170^b^	−41 ± 135^b^	342 ± 140^a^	276 ± 126^a^	−66 ± 99^a^
Stool characteristics						
Bristol Score	4.1 ± 1.0	4.2 ± 0.9	0.1 ± 1.0	3.9 ± 1.0	4.0 ± 0.9	0.1 ± 1.0
Stool frequency/day	1.4 ± 0.6	1.6 ± 0.9	0.1 ± 0.6	1.5 ± 0.7	1.5 ± 0.7	0.02 ± 0.3
Stool amount/day	1.6 ± 0.4	1.6 ± 0.4	−0.01 ± 0.5	1.5 ± 0.4	1.6 ± 0.5	0.05 ± 0.5

Data are presented as means ± SD; median (Q1; Q3). ^a^ *n* = 21; ^b^ *n* = 20. EN%, percentage of total energy intake; MET, metabolic equivalent of task; MUFA, monounsaturated fatty acids; IPAQ, International Physical Activity Questionnaire; PO, *Pleurotus ostreatus*; PUFA, polyunsaturated fatty acids; SFA, saturated fatty acids

### Compliance

The mean adherence to treatment was 99.1% ± 1.5% and 98.3% ± 2.3% in the PO and placebo group, respectively.

### Adverse effects

No significant differences in changes in gastrointestinal complaints were observed between the groups (*P* ≥ 0.05 for all; Additional File 1).

## Discussion

Contrary to our expectations, the present study showed that the daily consumption of 8.4 g of PO powder over 4 weeks had no effect on LDL-C, TC, HDL-C, TG, ApoA1, and ApoB concentrations in adults with moderately elevated LDL-C concentrations. However, reduction of cholesterol absorption as measured by validated surrogate markers was observed in a sex-dependent manner, with the reduction being especially prominent among females.

With regard to the lipid profile (Table 3), our results are in line with most findings of other RCTs providing either PO [17] or other edible mushrooms [40–42], which did not observe changes in LDL-C [17, 40, 42] and other lipids [TC [17, 40–42], HDL-C [17, 40–42], TG [40–42]] and in ApoA1 and ApoB [40, 42]. In fact, only the RCT of Hashemi Yusefabad et al. reported a reduction in LDL-C [41] and that of Schneider et al. in TG [17] compared to control treatment.

The lack of a reduction in LDL-C in our study (Table 3) raises questions regarding the appropriateness of the dose of fungal β-glucans provided by PO. In the present RCT, 8.4 g/d of powdered PO was provided as a granule, which is equivalent to approximately 84 g fresh PO [based on approximately 10% dry matter content [43]], providing 3 g/d β-glucans. This dose was selected based on the EFSA health claim on the cholesterol-reducing effects of oat β-glucans, which suggested that 3 g/d is needed for a reduction in LDL-C [4]. However, due to differences regarding β-glucan structure comparability might be limited. The amount of mushrooms consumed per day in the RCT of Hashemi Yusefabad et al. [41] was twice as high (16 g hot air-dried white button mushroom [*Agaricus bisporus*] powder) compared to the present study. Unfortunately, data on the content of β-glucans is lacking. Given that white button mushrooms generally contain less β-glucans than PO [44, 45], the dose alone may not adequately explain the lack of an effect in the present study. Other RCTs that found no treatment effect on LDL-C provided doses similar to those used in the present RCT [84 g fresh PO and *Agaricus bisporus* as part of a Mediterranean diet [42]; 10.4 g β-glucan-enriched extract from *Letinula edodes* with 3.5 g fungal β-glucans [40]] or even larger doses [30 g lyophilized PO equivalent to 300 g fresh mushrooms [17]].

It is rather unclear whether the intervention period employed in the present RCT (4 weeks) was too short to detect a treatment effect on LDL-C and other lipids. Other studies on edible mushrooms that had longer intervention periods (8 weeks) also failed to detect an effect [40–42]. Only one study found a decrease in LDL-C after 8 weeks [41], whereas another observed a decrease in TG already after 3 weeks [17]. The current study included participants with moderately increased LDL-C similar to Schneider et al. [17] and Morales et al. [40], whereas Hashemi Yusefabad et al. [41] included individuals with type 2 diabetes receiving medical treatment and Uffelman et al. [42] included healthy adults. The relatively high mean fiber intake (29 g/d) in the present study (Table 6) suggests a health-conscious population and a higher fiber intake can reduce TC and TG [46]. However, LM analysis showed that fiber intake at baseline did not affect the changes in LDL-C in this study.

The present RCT showed a reduction in the campesterol ratio and sitosterol ratio in females and in the 5α-cholestanol ratio in all subjects after adjustment for sex, suggesting that the daily intake of PO appears to lower cholesterol absorption. The present RCT observed that the effectiveness of PO in lowering cholesterol absorption appears to be sex-dependent. Yoshida et al. observed that women had higher serum concentrations of sitosterol and campesterol, whereas men had higher concentrations of lathosterol [47]. These sex-specific differences could be conceivably related to estrogen, which leads to a higher intestinal expression of Niemann-Pick C1-like 1 (*NPC1L1*) protein in female mice [48] and differences in bile-acid synthesis [49]. However, most women included in the present trial were postmenopausal, which limited the impact of estrogen on outcome parameters and mechanisms of action.

The present study found that BMI at baseline modulated the changes in markers of cholesterol absorption. Cross-sectional studies have suggested that an increased BMI was associated with reduced cholesterol absorption and increased cholesterol synthesis, whereas diet-induced weight loss increased cholesterol absorption and reduced cholesterol synthesis, indicating decreased cholesterol absorption and increased cholesterol synthesis in adults with obesity [50].

The present study suggests that PO treatment does not affect the synthesis of cholesterol (according to lathosterol ratio) and bile acids (according to 7α-hydroxycholesterol ratio), implying that PO mainly inhibits cholesterol absorption. However, we did not measure bile-acid concentrations in the feces. Bile-acid binding has been suggested as one of the main mechanisms by which PO-β-glucans lower cholesterol due to evidence from cereal β-glucans [5]. However, bile-acid binding should be followed by an increase in surrogate markers of bile-acid synthesis (e.g., 7α-hydroxycholesterol ratio), as shown after the intake of cholestyramine, a potent bile-acid binding resin that lowers cholesterol concentration in normocholesterolemic men [51]. An in vitro study by Belobrajdic et al. recently showed that the bile-acid binding capacity of β-glucans derived from fresh PO (22%) was lower than that of *Agaricus bisporus* (29%–36%) and oat β-glucans (36%) [45].

The present RCT showed that all participants exhibited an increase in serum ergosterol concentrations after PO treatment, which also confirms high treatment adherence rate. To the best of our knowledge, no human intervention study has yet investigated the effects of pure ergosterol intake on cholesterol metabolism. Gil-Ramirez et al. showed that ergosterol-containing extracts from *Agaricus bisporus* can modify the expression of cholesterol-related genes (e.g., *LDLR* and *SREBF2*) in Caco2 cells and mice models [52]. The explorative analysis of the expression of selected target genes involved in cholesterol metabolism conducted in the present study did not reveal any changes following the intervention (Table 5). Of course, gene expression in the liver would be the gold standard to investigate changes in cholesterol metabolism, however in RCTs with humans gaining liver samples is usually impossible. Given that the expression of genes e.g., *LDLR* and *HMGCR* in peripheral blood mononuclear cells is considered a valid surrogate marker for changes in cholesterol metabolism based on human intervention studies [53], any potential changes should have been detectable. The lack of treatment effect in the present study, however, is consistent with the lack of treatment effect on plasma LDL-C and other measured lipids. One study in mice showed that providing dietary fiber from PO simultaneously with a high-cholesterol diet modulated cholesterol-related gene expression in the liver, similar to simvastatin and ezetimibe, but without any effects on plasma cholesterol concentrations (LDL-C, TC, HDL-C) [54].

One strength of the present study is the selection of outcome parameters, including NCS, to investigate the possible mechanisms of action of PO in addition to the lipid profile. Moreover, assessment of dietary intake and physical activity indicated high adherence to lifestyle instructions, thereby limiting the confounding effects. Another strength is its double-blind, placebo-controlled design, which was implemented by incorporating PO in granule form. This approach was highly accepted by our participants as shown by the high compliance with both interventions, as well as the increase in serum ergosterol concentrations in the PO group. The unequal sex distribution between PO and placebo group is a clear limitation, considering the apparently substantial impact of sex on cholesterol absorption. However, owing to the relatively small sample size, our opportunities to conduct more statistically robust subgroup analyses e.g., subgroups by sex are limited. Moreover, measuring fecal bile-acid excretion would have been valuable and should be considered in future studies.

## Conclusions

In conclusion, the regular consumption of PO powder for 4 weeks (8.4 g/d, providing 3 g of fungal β-glucans daily) has no impact on LDL-C concentrations in adults with moderately elevated LDL-C concentrations. Nevertheless, a post-hoc analysis indicates a sex-dependent reduction in cholesterol absorption by PO consumption, especially in females. Overall, our findings suggest that PO might be able to lower cholesterol absorption and may therefore have the potential to beneficially modulate cholesterol metabolism.

## Supplementary Information

Supplementary Material 1

## Data Availability

Data described in the manuscript will not be made available as the participants were assured in the informed consent form that personal data will not be disclosed to third parties.

## Abbreviations

ACTB: β-actin
ASCVD: Atherosclerotic cardiovascular diseases
ApoA1: Apolipoprotein A1
ApoB: Apolipoprotein B
BMI: Body mass index
CI: Confidence interval
Cq: Quantification cycle
CYP7A1: Cytochrome P450,family 7,subfamily A, polypeptide 1
EFSA: European Food Safety Authority
GAPDH: Glyceraldehyde-3-phosphate dehydrogenase
GC-FID: Gas chromatography coupled with a flame ionization detector
GC-MS-SIM: Gas chromatography with coupled mass spectrometry in the selected-ion-monitoring mode
HDL-C: HDL-cholesterol
HMGCR: 3-hydroxy-3-methylglutaryl-CoA reductase
IPAQ: International Physical Activity Questionnaire
LDL-C: Low-density lipoprotein cholesterol
LDLR: Low-density lipoprotein receptor
LM: Linear models
NCS: Noncholesterol sterols
PO: Pleurotus ostreatus
RT-qPCR: Real-time quantitative polymerase chain reaction
RCT: Randomized controlled trial
SREBF2: Sterol regulatory element binding transcription factor 2
TC: Total cholesterol
TG: Triglycerides

## Declarations

Written informed consent was obtained from all individual participants before inclusion in the study.
