# Supplementary material for: Effects of regular consumption of a β-glucan-rich oyster mushroom powder on cholesterol metabolism in adults with moderately elevated LDL-cholesterol concentrations: a double-blind randomized controlled trial
Source: Nutr Metab (Lond). 2026 May 3;23:53. doi: 10.1186/s12986-026-01122-3 (PMC13147704; doi:10.1186/s12986-026-01122-3)
Supplement: Supplementary file 1 — Supplementary Material 1 [file 12986_2026_1122_MOESM1_ESM.docx]

**Additional File 1**

**Table 1.** Adverse effects before and after treatment with PO or placebo

|  | **PO group** (*n* = 23) | | | **Placebo group** (*n* = 23) | | | ***P* value^a^** |
| --- | --- | --- | --- | --- | --- | --- | --- |
|  | *Week 0* | *Week 4* | ∆ | *Week 0* | *Week 4* | ∆ |  |
| Bloating | 1 (1–2) | 1 (1–2) | 0 (0–1) | 2 (1–3) | 2 (1–3) | 0 (-1–1) | 0.408 |
| Flatulence | 2 (2–2) | 2 (1–3) | 0 (0–0) | 2 (2–3) | 2 (2–3) | 0 (-1–1) | 0.784 |
| Abdominal pain | 1 (1–1) | 1 (1–1) | 0 (0–0) | 1 (1–1) | 1 (1–1) | 0 (0–0) | 0.782 |
| Constipation | 1 (1–1) | 1 (1–2) | 0 (0–0) | 1 (1–2) | 1 (1–2) | 0 (0–0) | 0.579 |
| Diarrhea | 1 (1–1) | 1 (1–2) | 0 (0–1) | 1 (1–1) | 1 (1–1) | 0 (0–0) | 0.112 |
| Liquid stool | 1 (1–1) | 1 (1–2) | 0 (0–0) | 1 (1–1) | 1 (1–2) | 0 (0–0) | 0.658 |
| Hard stools | 1 (1–2) | 1 (1–1) | 0 (0–0) | 1 (1–2) | 1 (1–2) | 0 (-1–0) | 0.874 |
| Fullness | 1 (1–2) | 1 (1–1) | 0 (-1–0) | 1 (1–2) | 1 (1–2) | 0 (0–0) | 0.316 |
| Bowel noises | 1 (1–2) | 1 (1–2)^b^ | 0 (0–0)^b^ | 1 (1–2) | 1 (1–2) | 0 (0–1) | 0.839 |
| Nausea | 1 (1–1) | 1 (1–1) | 0 (0–0) | 1 (1–1) | 1 (1–1) | 0 (0–0) | 0.681 |

Data are presented as median (25th–75th percentile). ^a^ Comparison of the changes between the groups using non-parametric Wilcoxon rank-sum test; ^b^ *n* = 22 due to missing data from one participant. Symptom intensity was self-rated by participants on a 5-point Likert scale: 1 = “no complaints,” 2 = “mild complaints,” 3 = “moderate complaints,” 4 = “severe complaints,” and 5 = “very severe complaints.” PO*, Pleurotus ostreatus*.
